# CagA-positive Helicobacter pylori infection is not associated with decreased risk of Barrett's esophagus in a population with high H. pylori infection rate

**DOI:** 10.1186/1471-230X-6-7

**Published:** 2006-02-16

**Authors:** Angel Ferrández, Rafael Benito, Juan Arenas, María Asunción García-González, Federico Sopeña, Javier Alcedo, Javier Ortego, Ricardo Sainz, Angel Lanas

**Affiliations:** 1 Gastrointestinal Oncology Unit, Service of Digestive Diseases, Hospital Clínico "Lozano Blesa", Zaragoza, Spain; 2 Service of Microbiology, Hospital Clínico "Lozano Blesa", Zaragoza, Spain; 3 Unidad Mixta de Investigación, University of Zaragoza, Spain; 4 Department of Pathology, Hospital Clínico "Lozano Blesa", Zaragoza, Spain

## Abstract

**Background & aim:**

The role that H. pylori infection plays in the development of and Barrett's esophagus (BE) is uncertain. We tested the hypothesis that infection with cagA+ Helicobacter pylori strains protects against the development of BE.

**Methods:**

We studied 104 consecutive patients, residents in an area with a high prevalence of H. pylori infection, with BE and 213 sex- and age-matched controls. H. pylori infection and CagA antibody status were determined by western blot serology.

**Results:**

H. pylori prevalence was higher in patients with BE than in controls (87.5% vs. 74.6%; OR. 2.3; 95% CI: 1.23–4.59). Increasing age was associated with a higher prevalence of H. pylori (p < 0.05). The prevalence of CagA+ H. pylori serology was similar in patients with BE and controls (64.4% vs. 54.5%; NS). Type I H. pylori infection (CagA+ and VacA+) was similar in patients with BE and controls (44.2% vs. 41.3%; NS). Logistic regression analysis identified alcohol (O.R. 7.09; 95% CI 2.23–22.51), and H. pylori infection (OR: 2.41; 95%CI: 1.20–4.84) but not CagA+ serology as independent factors.

**Conclusion:**

Neither H. pylori infection nor H. pylori infection by CagA+ strains reduce the risk of BE in a population with high prevalence of H. pylori infection.

## Background

The incidence of adenocarcinoma of the esophagus has dramatically increased during the last 2 decades [1-3] and so has the incidence of Barrett's esophagus (BE), one of the most important risk factors for esophageal adenocarcinoma [4,5]. Despite the fact that BE is a predisposing factor, only 0.5% of the patients with BE will develop adenocarcinoma per year [6], meaning that factors that contribute to this development are unknown.

Helicobacter pylori is the major cause of peptic ulcer disease, and it is also implicated in the pathogenesis of adenocarcinoma of the distal stomach and gastric lymphoma, especially the mucosa-associated lymphoid tissue-type lymphoma (MALT lymphoma). The relationship between corpus gastritis, H. pylori infection, reflux oesophagitis and Barrett's esophagus is complex and not fully understood. It has been hypothesized that the presence of H. pylori infection or corpus gastritis may have a protective effect against GERD because of reduced gastric acid output [7-10].

An important virulence marker associated with duodenal ulcer disease is cagA, encoding the cytotoxin-associated gene protein (CagA) [11]. However, the effect of CagA+ strains in the development of Barrett esophagus might be in opposite direction to that observed in peptic ulcer disease. When compared to controls the prevalence of CagA+ H. pylori strains decreased with severity of complications of GERD, suggesting a protective factor of CagA+ strains in patients with Barrett's esophagus and its complications [12-15].

If this hypothesis is correct, H. pylori eradication should worsen the condition. However to add more confusion to this scenario, two recent studies have shown that H. pylori eradication does not influence relapse rates in GERD [16] and it may be even beneficial on symptomatic relapse in mild GERD [17]. It has been also suggested that gastritis and not H. pylori infection is associated with Barrett's esophagus. In fact, endoscopic diagnosis of reflux esophagitis or Barrett's esophagus is less common in patients with corpus gastritis [18]. A number of studies have examined the relationship between H. pylori infection and Barrett's esophagus. However, these studies are limited by small number of patients, the lack of appropriate control population and the low incidence of H. pylori in the general population.

The aim of this study was to determine whether infection with CagA+ Helicobacter pylori strains was associated with a lower risk of Barrett's esophagus development in an area with a high prevalence of H. pylori infection [13].

## Methods

### Patients

Since 1999 the Service of Digestive Diseases at the Hospital Clínico Lozano Blesa is collecting data of all the patients with endoscopic and pathologic diagnosis of Barrett's esophagus. Such register includes not only patients diagnosed with BE after that date but also all the available patients diagnosed since 1972, when the Section of Digestive Endoscopy was created. The Hospital Clínico Lozano Blesa comprises an area of about 250,000 inhabitants in the metropolitan area of Zaragoza. In the last two decades such population has been very stable with a slight increment. In Spain almost 98% of the people belong to the National Health Service that provides free Health care to all its members. The service of Digestive Diseases and the endoscopy unit are the only gastrointestinal facilities in that area, thus controlling about 98% of the patients with gastrointestinal diseases.

For the purpose of this study, control population was enrolled at the Blood donation facility in the Service of Hematology at the Hospital Clínico Lozano Blesa. Blood donors were asked to participate in the study. To be consider a blood donor in Spain, people must be at least 18 years old and no older than 75 and have no major pathologies. After informed consent was given, a simple health questionnaire completed prior blood extraction, with special attention to current and previous gastrointestinal diseases (mainly GERD related symptoms), previous and current medication. Patients with clinical symptoms of gastroesophageal reflux diseases or gastrointestinal symptoms as well as patients taking proton pump inhibitors were excluded from the control group.

Cases were consecutively enrolled from January 2000 to December 2002. After Barrett's esophagus was histologically diagnosed, patients were asked to participate in the study. After informed consent was obtained a complete health questionnaire was fulfilled including demographical data, prior and current own and familial medical conditions with special attention to gastrointestinal diseases and prior and current medications. Exclusion criteria were: inability of demonstrating Barrett's esophagus in the pathology examination, patients with prior gastrectomy, patients with a previous H. pylori eradication, and patients either with active or past gastric or duodenal ulcer were also excluded. In order to have a correct age-matching with the control population, patients under 18 years old and over 80 years old were not included in this study.

Smoking and alcohol intake habits were assessed by personal questionnaires as well as the socio-economical status. The proportion between rural- and urban-living individuals and socio-cultural status, were similar in both groups.

The study protocol was approved by the Institutional Review Board at the University Hospital of Zaragoza and all patients and controls gave informed consent to the study, which was conducted in accordance with the Ethical & Clinical Assays Committee.

### Endoscopy protocol

Endoscopy was performed in a fashioned way using either the Olympus GIF-100 (Olympus, Barcelona, Spain) video-endoscope or the Fujinon EG-300HR (Fuji, Madrid, Spain) video-endoscope. After the patient was sedated upper endoscopy was performed in a standard way and all the pathology observed was recorded.

The endoscopic grading of esophageal lesions was performed according the Los Angeles Classification [19]. Barrett's esophagus was endoscopically suspected when a reddish epithelium was seen above the gastroesophageal junction. Esophageal biopsies were obtained from the Barrett epithelium in two of the four quadrants and every two centimeters. Short segment Barrett esophagus was defined as columnar epithelium not longer than 2 cms and biopsies were taken every centimeter in such cases. Special attention was focused in the location of the squamo-columnar junction and the esophago-gastric junction with the purpose of identifying endoscopically the presence of suspicious ectopic mucosa. In order to avoid misinterpretation of short segment Barrett esophagus with carditis, biopsies were always taken from tongues of ectopic mucosa penetrating proximally into the esophagus. The presence or absence of hiatal hernia was always recorded and forward and retroflexed views were performed. Any suspicious lesion other than esophageal was informed and biopsied for study.

Neither rapid urease tests nor gastric histology were part of the study protocol and they were only collected in case of concomitant gastric or duodenal lesions.

### Histological Analysis

All biopsy specimens were fixed in Hollande's fixative and stained with H&E for interpretation by a single experienced pathologist (JO). Barrett's esophagus was defined as the presence of specialized columnar epithelium with acid mucin-containing globet cells in the esophagus. As mentioned previously, only patients with confirmed intestinal metaplasia in the histological analysis were considered as having Barrett's esophagus.

### H. pylori infection and strain analysis

**Figure 1:**
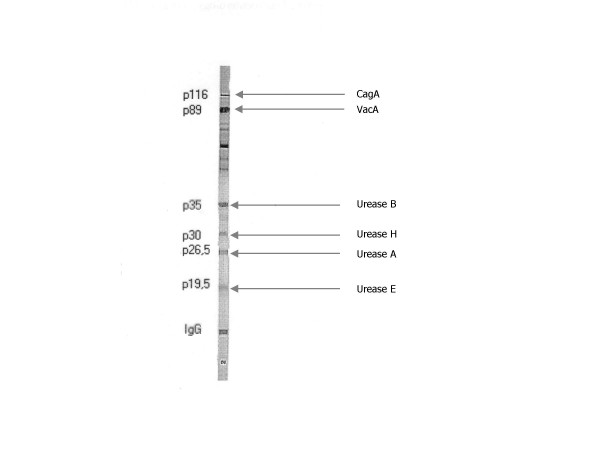
Western Blot for H. pylori infection.

The CagA status was determined in plasma by western blot with a commercial kit (Bioblot Helicobacter, Biokit SA. Barcelona, Spain), which has been validated in our area in previous studies [20-22]. This test determines the presence/absence of protein bands of 116 kDa (CagA), 89 kDa (VacA), 35 kDa (Urease B), 30 kDa (Urease H), 26.5 kDa (Urease A) and 19.5 kDa (Urease E). Any one band at 116 kDa, 89 kDa or 35 kDa was considered as positive for H. pylori infection. Any two bands from 30 kDa, 26.5 kDa or 19.5 kDa were also considered as positive for H. pylori infection as shown in Figure 1.

### Statistical analysis

Based in previous studies [20-22], the sample size was calculated using estimate prevalence of H. pylori of 70% in controls and a 20% reduction in patients with Barrett's esophagus (power = 0.80; alpha = 0.05). We analyzed differences between groups regarding H. pylori and its strains status, alcohol and smoking. We first made a bi-variate analysis for each risk factor separately using the chi-square test (with the Yates correction in variables with only two categories) and the Fisher test in tables with expected low rates. For multivariate analysis we performed a logistic regression model introducing gender and age to correct deviations due to theses two factors. We used BMDP software (BMDP Statistical Software) for the estimation of the logistic regression models. Odds ratio (OR) and their 95% confidence intervals (CI) were used to study the influence of the different risk factors in the development of Barrett's esophagus.

## Results

### Demographic characteristics and endoscopic and pathological findings

A total of 104 (82 male, 22 female) patients with Barrett's esophagus and 213 sex- and age-matched controls were included in the analysis. Demographic characteristics are shown in Table 1.

Short segment Barrett' esophagus was found in 67 (64.4%) patients and long segment in 37 (35.6%) patients. Seventy-five (72.1%) of the patients presented hiatal hernia.

When dysplasia was analyzed, 11 patients (11%) presented advanced pathology: high-grade dysplasia in 10 cases and adenocarcinoma in 1 case.

### Helicobacter pylori infection

In this population, the prevalence of H. pylori infection in patients with BE was greater than that found in controls (87.5% vs. 74.6% respectively; p = 0.013). The prevalence of CagA+ H. pylori serology was similar in patients with Barrett's esophagus and controls (64.4% vs. 54.5% respectively; p = 0.118). The presence of both CagA and VacA antibodys in serum, which defines a more virulent (type I) strain of H. pylori infection, was also similar in patients with Barrett's esophagus and controls (44.2% vs. 41.3% respectively; p = 0.710). Table 1 shows both H. pylori infection and environmental factors in patients with Barrett's esophagus and controls.

**Figure 2:**
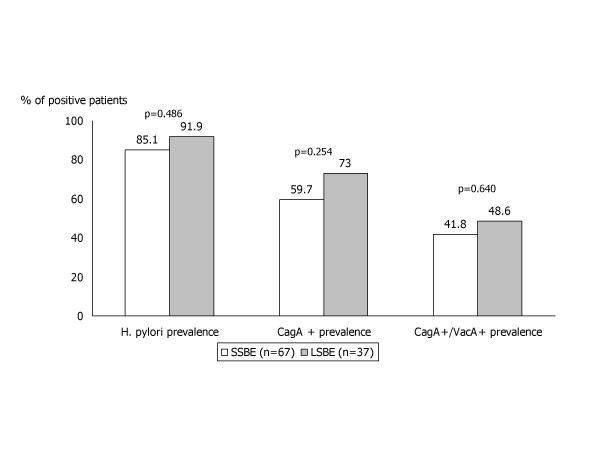
H. pylori infection related to age.

We also analysed the prevalence of H. pylori infection depending on the length of the Barrett's esophagus. No differences were found between short segment and long segment Barrett's esophagus regarding H. pylori status, cagA and VacA serologies as shown in Figure 2.

When combined with other environmental factors, logistic regression analysis identified H. pylori infection (O.R. 2.41; 95% CI 1.20–4.84), and alcohol intake (O.R. 7.09; 95% CI 2.23–22.51) but not or CagA + serology as independent factors associated with Barrett's esophagus.

**Figure 3:**
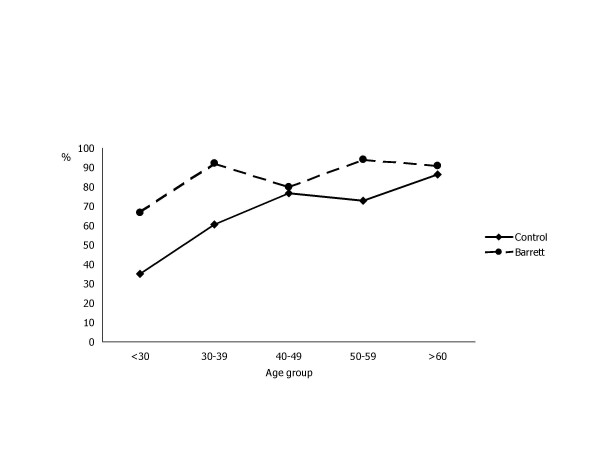
H. pylori status according to the length of Barrett's esophagus.

The prevalence of H. pylori infection according to age is shown in Figure 3. Regardless of the group, increasing age was associated with a higher prevalence of H. pylori (p < 0.05). We then decided to evaluate the influence of age in the relationship between Barrett's esophagus and H. pylori infection including cagA and vacA strains. There is a trend to a higher prevalence of H. pylori infection in the age group between 30 and 39 years in patients with Barrett's esophagus compared to controls (p = 0.064). Statistical differences are observed when CagA (p = 0.038) and VacA (p < 0.001) strains were considered independently. We then performed logistic regression considering the interaction between H. pylori and age (stratified as >40 and <40 years). The interaction was not statistically significant (data not shown) and the logistic regression model was similar to that obtained in the logistic regression without age stratification.

We also analyzed the influence of H. pylori infection on the development of dysplasia. However, the small number of patients with dysplasia did not allow us to make any statistic analysis.

## Discussion

Since the forties of the last century, hospitalization and mortality rates for H. pylori-associated duodenal ulcers have decreased. Conversely, those for GERD and esophageal adenocarcinoma, as well as Barrett's esophagus incidence have increased during that same period [23]. Thus, relationship between H. pylori infection and the development of GERD has been subject of study and controversy. Some studies have shown no causal association while others have suggested a possible protective role of H. pylori infection. The development of corpus gastritis and the virulence of the strains are different proposed mechanisms to explain such protection. It has been proposed that patients with H. pylori-induced gastric atrophy present a decrease in gastric acidity and subsequently a lower incidence of GERD [7-10]. Moreover, the presence of H. pylori is also likely to increase the efficacy of PPIs and conversely, the eradication of the bacteria decreases the drug effect. Our study was not designed to evaluate the mechanisms underlying such relationship, and the lack of a protocol-driven sampling of multiple gastric biopsies in our study, does not allow us to establish any relationship between H. pylori infection, gastric atrophy and Barrett's esophagus. This study was specifically designed to investigate whether gastric infection with CagA+ H. pylori strains was a protective factor for the development of Barrett's esophagus. To do this we have used an epidemiological approach and we found a greater prevalence of H. pylori infection in patients with Barrett's than that found in controls, and a similar proportion of CagA+ H. pylori strains in patients and controls.

Previous studies showed that H. pylori was less prevalent in patients with gastroesophageal reflux disease (GERD) than in control subjects [24-26].

Moreover, in the meta-analysis by Gisbert et al [25], the prevalence of H. pylori infection in patients with Barrett's esophagus was lower than the incidence in controls (28% vs. 45%. Odds Ratio: 0.6; 95% CI: 0.48–0.76). On the other hand, other studies have shown similar results to ours with higher incidence of H. pylori in patients with Barrett's esophagus [27].

Our findings strongly support that the presence of H. pylori is not associated with a decreased incidence of Barrett's esophagus in our population. The question now is why we have seen such differences in our study compared to other studies. This is far to be completely understood, but there are several aspects that may explain the differences between these studies. The characteristics of the studied population and the study design could be responsible for some of these differences. Our study is one of the larger case-control studies aimed to investigating the effect of H. pylori infection in patients with Barrett's esophagus. To our knowledge only the study by Lord et al [10] and the one by Weston et al [28] included more patients with Barrett's esophagus. However, in the study by Weston the control group only included GERD patients, who are obviously very different to our control group. We believe that GERD patients are not an appropriate control group in this setting, since both conditions are linked by similar pathogenic mechanisms. In the Lord study the results could be different because controls were patients that had undergone endoscopy meaning they were not asymptomatic. They also found that H. pylori was more prevalent in the control group, what could be explained (at least partially) by the selection of a symptomatic control group, and consequently with a higher possibility of being infected with H. pylori.

Choosing the correct control population is a key issue to asses the relationship between H. pylori and Barrett's esophagus. It could be argued that blood donors could also represent a biased population since not everyone is eligible as a donor. However we think that they represent an appropriate control population, since they were healthier and closer to the general population than those included in other studies, which were patients with GERD or those undergoing endoscopy due to gastrointestinal symptoms. Moreover, we excluded those people with GERD symptoms and those taking PPIs. Although we did not perform endoscopic procedures in this population, it must be outlined that there is a high predictive value of the presence of GERD when heartburn is the dominant or exclusive symptom [29].

Furthermore, the incidence of H. pylori is very different from one region to another [26]. In our area this incidence is very high, as we have shown previously [13]. Such a high incidence could mask a potential, weak, effect of H. pylori on the risk of Barrett's esophagus, but, on the other hand, it could be also expected that a real effect of H. pylori infection or virulent H. pylori strains on the development of Barrett's esophagus could be more easily shown in areas with high incidence of the infection in the general population. On the contrary, we found that H. pylori infection was more frequent in patients with Barrett's esophagus than in our control population, and that infection with the more virulent strains of H. pylori infection was not associated with a decreased risk for the development of Barrett's esophagus. In fact, the prevalence of CagA+ strains was similar in H. pylori positive patients and controls, although a trend toward a greater prevalence of virulent strains was seen in patients with Barrett's esophagus. In general, the prevalence of CagA+ strains obtained in different studies are lower than that found in our study, however a great variability is observed, with prevalences ranging from 13.3% to 82% [12,26,30]. In some way, it is surprising that H. pylori infection was greater in patients than in controls, indicating that there was a trend towards an increased frequency of non virulent strains (CagA negative) in controls that in cases. In any case, all these data indicate that CagA+ H. pylori strains do not seem to play an important role in this condition among our population and that other factors may be more important for the development of Barrett's esophagus. These additional factors could be intrinsic to the host (i.e. genetic factors) and extrinsic. In fact, it has been suggested that there may be a genetic predisposition to the development of reflux in families of patients with Barrett's esophagus, although a specific genetic defect has not been found [31,3].

For uncomplicated reflux esophagitis, environmental factors seem important. Smoking and dietary factors play an indirect role in the pathophysiology of GERD. Alcohol can decrease LES pressure and in moderate amounts impairs the normal acid clearance in supine position. The effect of smoking is less clear: it can increase the number of reflux episodes, decrease LES pressure and increase esophageal acid exposure [34]. We analyzed the influence of such external factors as alcohol and smoking in this population and an alcohol intake of more than 80 grams per day increases the risk of Barrett's esophagus. However, smoking does not seem to have any influence on the development of this condition independently of the amount of cigarettes smoked a day. However, these results have to be taken with caution since the sample size of the study was not calculated based on these aspects.

One factor that has been linked to the development and severity of GERD and Barrett's esophagus is the presence of a hiatal hernia. We found that 72% of the patients presented hiatal hernia. These results are similar to those reported by Arvidan et al [35,36] who found a similar prevalence of hiatal hernia in patients with Barrett's esophagus (65%). Thus, Barrett's esophagus occurred more frequently among subjects with hiatus hernia than in controls (65% vs. 24%). Moreover, the size of hiatal hernia increased not only the risk of Barrett's esophagus but also the risk for adenocarcinoma and severe forms of GERD [35,36].

One of the potential weakness of the study is the use of serology for the diagnosis of H. pylori infection. However, we needed a non-invasive diagnostic test to perform in controls and serology was the only feasible test to detect the presence of CagA positive H. pylori strains, since other non-invasive techniques as urea breath test could not provide such information. Furthermore, previous studies in populations from the same area have demonstrated that serology had an excellent correlation with the results obtained by either the urea breath test or the rapid urease test [20,21], and a good method to asses the presence or absence of CagA and VacA antibodys. Moreover, the control group was enrolled from the blood donation facility making a blood-based test very convenient because it can be performed very easily in healthy controls in which an endoscopic procedure would be hardly justifiable and perhaps unethical. We did not take any biopsy to asses *Helicobacter pylori *status and it could be argued that serological methods to asses the presence and strain of *Helicobacter pylori*, is not sufficient to not consider gastric histology a valuable tool for strain genotyping or evaluation of extension/severity of gastritis. However, the biopsy protocol for patients with Barrett's esophagus in our hospital does not include routine gastric sampling for Helicobacter pylori investigation if no other lesion is found during endoscopy. Furthermore, performing endoscopies in controls to assess *Helicobacter pylori *status through biopsies would have been difficult to explain and could be even considered unethical.

The exclusion of BE cases with active or past peptic ulcer could be considered a selection bias towards patients with less virulent strains compared to controls. However, most of the patients with peptic ulcer history in our region are infected with *Helicobacter pylori *and including such cases would have represented a higher selection bias. Moreover, excluding peptic ulcer patients reduces the chance that individuals in the control group had a better chance to be colonized by cagA-negative *Helicobacter pylori *strains compared to individuals in the study group.

Another potential weakness of our study is the diagnosis of Barrett's esophagus. We have used endoscopic and pathologic criteria. However, from a histological point of view, it is impossible to distinguish between intestinal metaplasia in the esophagus from that at the gastro-esophageal junction (GEJ). This is particularly important in patients diagnosed with short segment Barrett's esophagus. Some authors have used specific staining cytokeratin markers CK7 and CK20 to differentiate the two types [37]. Although we tried to avoid biopsies at the GEJ there is not a way to unequivocally exclude patients with intestinal metaplasia of the GEJ.

If our study would have included many cases misdiagnosed as SSLB we should have observed a lower prevalence of H. pylori infection in the LSBE group compared to the SSBE, similar to what we observed in cases versus controls. However, we did not find such differences suggesting that the diagnosis of Barrett' esophagus was correct or, at least, that if we included few misdiagnosed patients, they did not have a major influence on the results.

In conclusion, this study demonstrates that, neither H. pylori infection nor H. pylori infection by cagA+ strains decrease the risk of Barrett's esophagus in a population with high prevalence of H. pylori infection. Further research is needed to identify other factors like environmental or genetics that may play a role in Barrett's development into cancer.

## Abbreviations

SSBE: short-segment Barrett's esophagus; LLSB: long-segment Barrett's esophagus.

## Competing interests

The author(s) declare that they have no competing interests.

## Authors' contributions

Angel Ferrández selected the patient and control population, co-designed the database, helped to the design of the study, and drafted the manuscript.

Rafael Benito processed the blood samples and analysed the H. pylori status and strains.

Juan Arenas selected the patient and control population, helped to the design of the study and co-designed the database.

María Asunción García-González helped with the analyses of H. pylori strains.

Federico Sopeña helped with the selection of patients and adquisition of data from patients database.

Javier Alcedo helped with the selection of patients and adquisition of data from patients database.

Javier Ortego performed all the histologic analyses

Ricardo Sainz helped with the design of the study.

Angel Lanas designed the study and helped with the draft of the manuscript.

## Pre-publication history

The pre-publication history for this paper can be accessed here:



## Figures and Tables

**Table 1:** Demographics and risk factors

		**Control (n = 213)**		**Barrett's (n = 104)**		**OR**	**95% C.I.**	**p**
		n	%	n	%			
**Age**		53.37 ± 16.01		53.96 ± 16.25				0.995
**Gender**	Male	147	69.0	82	78.8	1.67	0.96–2.91	0.089
	Female	66	31.0	22	21.2			
	M:F ratio	7.9:2.1		7:3				
**Alcohol**	No or <80 g/day	209	98.1	90	86.5	8.13^b^	2.60–25.37	0.000
	>80 g/day	4	1.9	14	13.5			
**Smoking**	No	130	61.0	54	51.9			
	Yes	66	31.0	32	30.8			0.039
	Former	17	8.0	18	17.3			
**H. Pylori**	Positive	159	74.6	91	87.5	2.38	1.23–4.59	0.013
**CagA +**	Positive	116	54.5	67	64.4	1.51	0.93–2.46	0.118
**VacA +**	Positive	99	46.5	59	56.7	1.51	0.94–2.42	0.111
**Type1**^a^	Positive	88	41.3	46	44.2	1.13	0.70–1.81	0.710

^a^Type1: CagA+ vacA+

^b^OR of Barrett's esophagus for >80 grams of alcohol per day vs. <80 gr/day
